# Anti-Epstein–Barr virus antibodies in Beijing during 2013–2017: What we have found in the different patients

**DOI:** 10.1371/journal.pone.0193171

**Published:** 2018-03-01

**Authors:** Jingtao Cui, Wenjuan Yan, Shaoxia Xu, Qiaofeng Wang, Weihong Zhang, Wenjing Liu, Anping Ni

**Affiliations:** Department of Clinical Laboratories, Peking Union Medical College Hospital, Chinese Academy of Medical Sciences and Peking Union Medical College, Beijing, China; United Arab Emirates University College of Medicine and Health Sciences, UNITED ARAB EMIRATES

## Abstract

**Background:**

Epstein**–**Barr virus (EBV) is associated with nasopharyngeal carcinoma (NPC) which is prevalent in South China, and its association with systemic lupus erythematosus (SLE) or other autoimmune diseases has not been studied in the mainland of China. The EBV serological tests have been performed on patients with various diseases or manifestations for years at our institution and their values need to be evaluated.

**Methods:**

For routine medical purposes, anti-EB viral capsid antigen (VCA) IgG, IgA and IgM antibodies, anti-EBV diffuse early antigen (EA-D) IgA antibodies, and anti-EBV nuclear antigen-1(EBNA-1) IgG antibodies were tested with commercial enzyme-linked immunosorbent assay (ELISA) in patients visiting Peking Union Medical College Hospital between 2013 and 2017. The test results were analyzed in this retrospective study.

**Results:**

There were a total of 11122 serum samples available to be tested in the study. As indicators of past EBV infection, the prevalence of VCA-IgG/EBNA1-IgG were 66.6%/58.5%, 84.3%/78.8%, 92.9%/87.0% and 98.5%/95.4% in patients aged under 5 years, 6**–**10 years, 11**–**20 years and 21**–**30 years old, respectively, and these values maintained at this highest rate as age increased further. The prevalence of VCA-IgM, as a parameter of acute EBV infection, was 14.6%, 10.2%, 10.4%, 6.3% and 3.1% in patients aged under 5 years, 6**–**10 years,11**–**20 years, 21**–**30 years, 31**–**40 years old, respectively, and decreased to 2%~3% in older patients. Patients with elevated serum liver enzymes were more likely to have a higher prevalence of EA/D IgA antibody (*P* < 0.01) and young patients (≤30 years) with lymphadenopathy were more likely to have higher prevalence of VCA-IgM antibody (*P* < 0.01). The prevalence of VCA-IgA and EAD-IgA were 87.0% and 59.2% in NPC patients, respectively, and both were significantly higher (*P* < 0.001) than that in non-NPC patients. The prevalence of VCA-IgA was 45.4% and 25.6% in SLE patients and patients with other autoimmune diseases, respectively, which were significantly (*P* < 0.001) and mildly (*P* = 0.039) higher than their controls. In pediatric SLE patients between 6 and10 years old, the prevalence of VCA-IgG, VCA-IgA and EBNA1-IgG was 100%, 59.5% and 100%, respectively, all being significantly higher than the age (6-10y) related controls (*P*< 0.01). In the 705 cerebral spinal fluid (CSF) specimens, VCA-IgG, VCA-IgM, VCA-IgA and EAD-IgA were found to be positive in 12.1%, 0.15%, 0.25% and 0.25%, respectively. There were 157 paired specimens (CSF and serum were collected simultaneously) and VCA-IgG was identified as positive in 12.7% of the CSF and 100% of the serum specimens.

**Conclusions:**

Around 98% of Chinese patients were infected with EBV before 30 years of age and the highest rate of acute EBV infection were observed in patients under 5 years old. EBV infection was found to be associated with elevated serum liver enzymes, NPC and SLE. Acute anti-EBV antibody was valued for young patients with lymphadenopathy but limited value for CNS neuropathy.

## Introduction

Epstein**–**Barr virus (EBV) is a family member of the *Herpesviridae* family. Most people become infected with EBV at some point in time in their life and the majority of infections occur in children and teenagers [1]. Primary infection with EBV is mainly symptomless, however, latent infection can be present for life. The classical manifestations of EBV infection, if patients have, are fever, sore throat, lymphadenopathy, hepatitis, splenomegaly, etc. EBV infection is associated with various diseases, including infectious mononucleosis (IM), Burkitt’s lymphoma, Hodgkin’s disease, nasopharyngeal carcinoma (NPC) and gastric cancer [1, 2]. The etiology of autoimmune diseases, including systemic lupus erythematosus (SLE), remains unclear, and may involve genetic, environmental or other factors. Viral infections certainly contribute to the environmental factors. Several studies have reported the association between EBV infection and SLE [3**–**6], or other autoimmune diseases [7**–**9]. Studies on the association between EBV infection and SLE in Chinese patients in Taiwan have also been performed and the results supported the correlation [10–11]. In the mainland of China, there are many patients with autoimmune diseases, however there have not been many studies to investigate the possible linkage. EBV serological tests have been performed as routine laboratory diagnostics for patients with various diseases or manifestations for years at our institution, and it may be the time to analyze their values.

## Subjects and methods

### Ethics statement

This retrospective study was approved by the Peking Union Medical College (PUMC) Hospital Ethics Committee (reference no. S-K320) and was waived the requirement for informed consent, because the blood and cerebral spinal fluid (CSF) samples were collected and tested for routine medical purposes. The patients’ identifications had been removed before the analysis of the test results.

### Study samples

For routine medical purposes, 11122 serum and 705 CSF samples from various Chinese inpatients and outpatients were collected and tested for anti-EBV antibodies at the PUMC Hospital in Beijing between September 2013 and July 2017. We conducted this retrospective study by analysis of the test results to determine the prevalence and risk factors that were associated with EBV infection.

Inclusion criteria were samples from patients who: were Chinese, had demographic information and had at least one parameter of anti-EBV antibodies for the laboratory testing.

Exclusion criteria were samples from patients who: were foreigners (as this study was designed only for Chinese patients), had no demographic information or had no laboratory test results of anti-EBV antibodies.

### Laboratory testing

Anti-EBV viral capsid antigen (VCA) IgG, IgA and IgM antibodies, anti-EBV diffuse early antigen (EA-D) IgA antibodies, and anti-EBV nuclear antigen-1(EBNA-1) IgG antibodies were tested with commercial enzyme-linked immunosorbent assay (ELISA) kits (Euroimmun Medical Diagnostics, Lübeck, Germany). ELISA was performed according to the manufacturer’s instructions. The absorbance was measured at a wavelength of 450nm and a reference wavelength of 630nm (Sunrise, Tecan Company, Austria). The signal-to-cutoff ratio (S/CO) of specimens ≥1.1 was considered as positive, < 0.8 as negative and ≥ 0.8 but < 1.1 was equivocal. Please refer to S1 File in the support information (anti-EBV.xlsx) for the details.

### Statistical analysis

EBV serological testing results were originally stored in our laboratory information system (LIS). These data were transformed from the LIS, sorted and analyzed in Microsoft Excel 2007 (Microsoft Corp., New York, NY). Statistical analysis was performed with IBM SPSS software (Version 21.0). *P*-values <0.05 were considered statistically significant. Pearson chi-square test with continuity correction and Fisher’s exact test (two-tailed) were used to compare the prevalence according to patient age, sex, diseases or manifestations.

## Results

### Total prevalence and age-related anti-EBV antibodies

A total of 11122 serum specimens were tested for anti-EBV antibodies between September 2013 and July 2017. The specimens were from 5721(51.4%) males and 5401 (48.6%) females. Patients’ ages ranged from 8 days after birth to 101 years (mean age, 39.0±19.9 years; median age, 40 years), the mean ages for males and females were 39.6±20.3 and 38.4±19.5 years, respectively.

As presented in Table 1, each of 11122 serum samples was tested for one to five parameters of anti-EBV antibodies, including VCA-IgG, VCA-IgM, VCA-IgA, EAD-IgA and EBNA1-IgG. Actual numbers of patients in each age group tested for each parameter could be calculated from the positive numbers divided by their percentages.

**Table 1:** Age related anti-EBV antibodies in all patients visiting the PUMC Hospital, Beijing, 2013–2017.

	VCA IgG Pos no. (%)	VCA IgM Pos no. (%)	VCA IgA Pos no. (%)	EA/D IgA Pos no. (%)	EBNA1 IgG Pos no. (%)
0–5y*	283(66.59)	65(14.57)	52(14.57)	26(7.12)	141(58.51)
6–10y	431(84.34)	55(10.24)	93(22.79)	39(9.18)	226(78.75)
11–20y	784(92.89)	123(10.41)	178(23.73)	95(12.20)	413(86.95)
21–30y	809(98.54)	88(6.25)	192(26.10)	120(15.33)	271(95.43)
31–40y	853(98.84)	40(3.06)	219(22.71)	123(11.80)	203(94.86)
41–50y	892(99.78)	36(2.76)	282(22.54)	171(13.13)	202(97.57)
51–60y	957(99.79)	37(2.62)	301(27.54)	184(15.79)	248(96.12)
61–101y	902(99.01)	29(2.03)	258(33.42)	146(18.36)	258(93.82)
Male	2905(94.23)	229(5.13)	864(25.62)	504(14.31)	938(86.93)
Female	3006(95.58)	244(5.35)	711(24.03)	400(12.76)	1024(87.90)
Total	5911(94.91)	473(5.24)	1575(24.87)	904(13.58)	1962(87.43)

* Actual numbers of patients in each (age) groups tested for each parameter could be calculated from the positive numbers divided by their percentages.

The overall prevalence of VCA-IgG, VCA-IgM, VCA-IgA, EAD-IgA and EBNA1-IgG were 94.9%, 5.2%, 24.9%, 13.6% and 87.4%, respectively. For males, the prevalence was 94.2%, 5.1%, 25.6%, 14.3% and 86.9%, respectively. For females, the prevalence was 95.6%, 5.3%, 24.0%, 12.7% and 87.9%, respectively. The prevalence of VCA-IgG/EBNA1-IgG, as indicators of past EBV infection, increased with age, and were 66.6%/58.5%, 84.3%/78.8%, 92.9%/87.0% and 98.5%/95.4% in patients aged under 5 years, 6**–**10 years,11**–**20 years and 21**–**30 years old, respectively, and remained at this highest rate as age increased further. In contrast, the prevalence of VCA-IgM, as a parameter of acute EBV infection, was 14.6%, 10.2%, 10.4%, 6.3% and 3.1% in patients aged under 5 years, 6**–**10 years,11**–**20 years, 21**–**30 years, 31**–**40 years old, respectively, and decreased to 2%~3% in older patients.

There was 1 patient with Burkitt’s lymphoma, 4 patients with gastric cancer, 17 with Hodgkin’s disease, 34 with infectious mononucleosis, and 483 with NPC in this study. Due to the large number of NPC patients with very high seroprevalence of EBV VCA IgA and EA/D IgA antibodies, the 483 NPC patients (mean age, 48.7 ± 13.0 years; median age, 49 years) were excluded from the total number of patients and the rest of the patients without NPC (non-NPC) were re-arranged into different age groups, as presented in Table 2. Table 2 was designed for two purposes, the first was to show the prevalence distribution closer to general patients, and the second was to re-arrange patients into different age groups (non-NPC patients) to create statistical controls.

**Table 2:** Age-related anti-EBV antibodies in all non-nasopharyngeal carcinoma patients visiting the PUMC Hospital, Beijing, 2013–2017.

	VCA IgG Pos no. (%)	VCA IgM Pos no. (%)	VCA IgA Pos no. (%)	EA/D IgA Pos no. (%)	EBNA1 IgG Pos no. (%)
0–5y	283(66.59)	65(14.57)	52(14.57)	26(7.12)	141(58.51)
6–10y	429(84.28)	55(10.28)	92(22.66)	39(9.20)	226(78.75)
11–20y	775(92.81)	123(10.49)	171(23.05)	89(11.54)	411(86.89)
21–30y	774(98.47)	88(6.40)	160(22.79)	96(12.80)	268(94.37)
31–40y	811(98.78)	39(3.07)	182(19.72)	97(9.67)	199(94.76)
41–50y	759(99.74)	35(2.98)	170(15.15)	109(9.24)	189(97.42)
51–60y	822(99.76)	31(2.43)	185(19.33)	108(10.44)	238(95.97)
61–101y	834(98.93)	24(1.77)	202(28.53)	101(13.93)	252(93.68)
Male	2613(93.62)	222(5.3)	608(19.70)	343(10.57)	911(86.60)
Female	2874(95.39)	238(5.37)	606(21.41)	322(10.71)	1013(87.78)
Total	5487(94.54)	460(5.36)	1214(20.52)	665(10.64)	1924(87.22)

### Anti-EBV antibodies in different patients or manifestations

Comparisons of anti-EBV antibodies in non-NPC patients with various symptoms who were 30 years old or younger are presented in Table 3. The prevalence of VCA-IgM was found to be significantly higher in patients with lymphadenopathy than that in total of ≤30 year old non-NPC patients (*χ*^*2*^ = 9.59, *P* = 0.002). The prevalence of EA/D IgA antibodies was also found to be significantly higher in patients with abnormal liver function (elevated serum liver enzymes) than that in total of ≤30 year old non-NPC patients (*χ*^*2*^ = 8.74, *P* = 0.003). In the fact, the overall prevalence of EA/D IgA was also found to be significantly higher in non-NPC patients of all ages (0-101y) with elevated serum liver enzymes than that in all non-NPC patients (27/146 vs. 665/6252, *χ*^*2*^ = 13.11, *P* = 0.000).

**Table 3:** Comparison of anti-EBV antibodies in ≤30 years old non-nasopharyngeal carcinoma patients with various symptoms at the PUMC Hospital, Beijing, 2013–2017.

	VCA IgG Pos no. (%)	VCA IgM Pos no. (%)	VCA IgA Pos no. (%)	EA/D IgA Pos no. (%)	EBNA1 IgG Pos no. (%)	*χ*^*2*^/*P*-value
FUO	534(83.57)	92(9.89)	106(18.06)	66(10.59)	319(79.35)	
Lymphadenopathy	119(88.15)	**32(16.41)**	18(16.98)	11(9.65)	38(82.61)	9.59/0.002*
Elevated serum liver enzymes	62(86.11)	18(13.33)	17(27.87)	**16(22.86)**	31(75.61)	8.74/0.003**
Total of ≤30y non-NPC	2261(88.49)	**331(9.38)**	475(21.52)	**250(10.82)**	1046(81.40)	

FUO: Fever of unknown origin

Non-NPC: Non- nasopharyngeal carcinoma

* Comparison of anti-VCA IgM antibodies between ≤30 year old non-NPC patients with lymphadenopathy and total of ≤30 year old non-NPC patients;

** Comparison of anti-EA/D IgA antibodies between ≤30 year old non-NPC patients with elevated serum liver enzymes and total of ≤30 year old non-NPC patients.

The seroprevalence of anti-EBV antibodies among various patients, including those with NPC, SLE, other autoimmune diseases (non-SLE) as well as CNS neuropathy are presented in Table 4. The prevalence of both VCA IgA and EA/D IgA in NPC patients were significantly higher than that in the non-NPC patients (*χ*^*2*^ = 550 or 373, *P* = 0.000). Among SLE patients, only the prevalence of VCA IgA was compared and a significant difference (*χ*^*2*^ = 34.8, *P* = 0.000) was observed between SLE and non-SLE patients. In pediatric SLE patients between 6 and 10 years old, the prevalence of VCA-IgG, VCA-IgA and EBNA1-IgG were 100%, 59.5% and 100%, respectively, all being significantly higher (*P*< 0.01) in pediatric SLE patients than in non-SLE pediatric patient.

**Table 4:** Comparison of anti-EBV antibodies in different patients visiting the PUMC Hospital, Beijing, 2013–2017.

	Mean±SD or Age group(yr.)	VCA IgG Pos no. (%)	VCA IgM Pos no. (%)	VCA IgA Pos no. (%)	EA/D IgA Pos no. (%)	EBNA1 IgG Pos no. (%)
NPC	48.67 ±12.96	424(100)	13(3.10)	361(86.99)	239(59.16)	38(100)
non-NPC	51–60y	822(99.76)	31(2.43)	185(19.33)	108(10.44)	238(95.97)
*χ*^*2*^/*P*-value				550.23/0.000*	**373.88/0.000**	
SLE(total)	24.81 ±15.13	233(100)	19(4.74)	79(45.40)	26(13.20)	130(99.24)
non-NPC	21–30y	774(98.47)	88(6.40)	160(22.79)	96(12.80)	268(94.37)
*χ*^*2*^/*P*-value				**34.80/0.000**		
SLE(6-10y) **	8.47 ±1.69	52(100)	1(2.00)	25(59.52)	7(14.89)	34(100)
non-NPC& non-SLE(6-10y) ***	8.12 ±1.46	377(82.50)	54(11.13)	67(18.41)	32(8.49)	192(75.89)
*χ*^*2*^/*P*-value		**9.52/0.002**		**34.02/0.000**		**9.02/0.003**
Other Autoimmune Diseases (Non-SLE)	37.13 ±19.89	366(95.31)	29(4.04)	75(25.60)	29(8.98)	149(91.41)
non-NPC	31–40y	811(98.78)	39(3.07)	182(19.72)	97(9.67)	199(94.76)
*χ*^*2*^/*P*-value				**4.27/0.039**		
CNS neuropathy	40.21 ±19.0	77(98.72)	**0(0/104)**	14(24.06)	7(13.46)	20(90.91))
non-NPC	41–50y	759(99.73)	**35(2.98)**	170(15.15)	109(9.67)	189(97.42)

* Results of comparison of VCA IgA between NPC patients (upper line) and non-NPC patients (lower line), same patterns were used for other comparisons in table 4.

**SLE(6–10y): SLE patients between 6 and 10 years old were chosen for comparison as there was only one SLE patient under 6 years old.

*** Non-NPC & non-SLE (6–10y): All 6–10 years old patients in whom NPC and SLE were excluded.

### Anti-EBV antibodies in CSF specimens

Of the 705 CSF specimens, 394 (55.9%) specimens were from male patients (mean age, 41.9±17.9 years) and 311 (44.1%) were from females (mean age, 40.5±17.9 years). The overall prevalence of VCA-IgG in CSF samples was 12.1% (54/445), and the sex-specific prevalence being 12.6% (32/253) for males and 11.5% (22/192) for females. VCA-IgM, VCA-IgA and EA/D IgA antibodies were tested to be positive in 0.15% (1/678), 0.25% (1/398) and 0.25% (1/393), respectively.

There were 157 pairs CSF and serum samples which were collected simultaneously, VCA-IgG antibody was identified as positive in 12.7% (20/157) of the CSF specimens and 100% (157/157) of the serum specimens. In the 20 pairs of samples with dual positives of VCA-IgG, 1 pair was found to have higher absorbance of VCA-IgG in the CSF than in the serum sample (Table 5).

**Table 5:** EBV VCA-IgG antibodies in the 157 paired samples from patients with various symptoms or diseases.

	Only Serum VCA-IgG Pos no.	Only CSF VCA-IgG Pos no.	Serum &CSF VCA-IgG Pos no.	Absorbs in Serum / Absorbs in CSF >1 Pos no.	Absorbs in Serum / Absorbs in CSF <1 Pos no.
CNS & peripheral neuropathy	95	0	15	14	1
FUO	8	0	2	2	0
Autoimmune diseases	11	0	0	0	0
Other diseases	6	0	0	0	0
Not indicated (from other hospitals)	17	0	3	3	0
Total	137	0	20	19	1

## Discussion

In France, EBV infection has decreased during the last 15 years, the proportion of seronegative patients increased from just over 50% during 2001**–**2005 to more than 60% during 2011**–**2015 period in patients < 10 years old [12]. Similarly in Japan, the prevalence of EBV infection in 5**–**7 year old was more than 80% in the early 1990s and decreased to 59% during 1995–1999 periods [13]. Xiong et al [14] reported that past EBV infection rates were still hold at high levels in China compared to the age-specific prevalence of anti-EBV antibodies in 1**–**10 years age health children in 2014, during which period the prevalence of VCA-IgG/EBNA-1 IgG antibodies were determined to be 77.4%/76.3% and 77.8%/75.5% in Beijing and Guangzhou, respectively. A study from Taiwan in 2007 [15] showed that the prevalence of VCA-IgG antibody was 88.7% in 5**–**7 years old normal children. Interestingly, our children patients had the same incidence of past EBV infection as the above mentioned health Chinese children, because the prevalence of VCA-IgG/EBNA1 IgG antibodies in our study were 66.6%/58.5% in patients under 5 years old, 84.3%/78.7% in patients of 6**–**10 years old, and with the average of 76.3% (714/936) and 69.5% (367/528) in patients of 0**–**10 years old. Regarding to adult patients, our prevalence of VCA-IgG in patients of 21**–**30 years old was 98.5%, and it was as same as the 98.4% in 20**–**29 year olds in the general population in Taiwan [15].

However, unlike past EBV infection, prevalence of EBV VCA-IgM, a parameter of acute EBV infection, was 12.2% (120/983) in 0**–**10 year old children patients in our study, which was much higher than the 1.6%-2.7%/ positive rate in 1**–**10 year healthy children without the EBV-related symptoms [14].

Clinical manifestation of fever of unknown origin (FUO) was closely associated with EBV infections [16, 17], Lymphadenopathy and elevated serum liver enzymes were also linked to EBV infections, especially in adolescents [18**–**20]. In our study, the highest frequency of acute EBV infections occurred in 30 years old or younger patients and lymphadenopathy was found to be associated with VCA-IgM antibody in these younger patients, indicating that acute EBV infection should be considered and serological tests may be performed to confirm when such patients visit doctors.

EA/D is an antigen expressed by EBV at the initiation of lytic replication and reactivation of the virus [21, 22]. As it was shown in Table 3, EA/D IgA, rather than VCA IgM was associated with elevated serum liver enzymes, thus we believe that the EBV related hepatitis is more likely to be a chronic rather than an acute process, and anti-EA/D IgA antibody may be expected to be an indicator of the process.

Compared to other parts of the world, the incidence of NPC is high in South China, where NPC occurs in 50/100,000 population [23]. EBV serum testing of VCA-IgA was diagnostically most valuable for NPC [24, 25], however, 4–24% of NPC patients remained negative for VCA-IgA [25]. The combined test of VCA-IgA and EBNA1-IgA antibodies using ELISA has been recognized as the optimal method for NPC screening, which showed 95.3% sensitivity and 94.1% specificity [26]. In our study, 87.0% of NPC patients were found to have serum VCA-IgA antibodies, suggesting that EBNA1-IgA test is needed to increase the sensitivity of NPC screening at our institution.

In the meta-analysis of 25 case control studies of EBV and SLE, Hanlon P et al [26] reported that the seroprevalence of EBV VCA-IgG, IgA, and EA/D IgG antibodies, though possibly not anti-EBNA-1, were significantly higher in cases than controls. However, the study by Chougule D et al [6] showed that the antibodies levels of EBV VCA-IgG, VCA-IgM, and EBNA-1 IgG in SLE patients were significantly higher than that in healthy controls. Furthermore, a correlation was also shown between EBV infection and disease activity of SLE [27]. Among all SLE patients in our study, both EBV VCA-IgG and EBNA1-IgG antibodies could not be compared with the controls due to the high percentage of antibodies present in all adult patients, but we still found a significantly higher (*P* < 0.001) prevalence of serum VCA-IgA among all SLE patients versus the age matched controls. When the data of pediatric SLE patients of 6**–**10 years old were analyzed separately, the observations were different. Significant differences were observed not only in the prevalence of VCA-IgA (*P* < 0.001), but also in the prevalence of VCA-IgG and EBNA1-IgG antibodies (*P* < 0.01) between the 6**–**10 years old SLE patients and controls. The overall EBV past infection with common chronic active infection (as high prevalence of VCA IgA) in pediatric SLE patients in our study provides, on another hand, evidence for the reports [6, 28] that EBV infection may pay a big role or being a trigger in the pathogenesis of SLE. For other autoimmune diseases, it seemed that the association with EBV was not as strong as that with SLE patients (*P* = 0.039).

The relationship between EBV infection and central nervous system (CNS) diseases was indeterminate, some studies [29, 30] supported it, and others [31, 32] did not. Serologic value seemed to be limited for diagnosis of EBV related CNS diseases in our study. Firstly, there were 107 patients with CNS neuropathy in the study, 104 were tested for serum VCA-IgM antibodies and none were positive (as shown in Table 4). Secondly, of 705 CSF specimens, only 0.15% (1/678), 0.25% (1/398) and 0.25% (1/393) were tested to be positive for EBV VCA-IgM, VCA-IgA and EA/D IgA, respectively.

In the 157 paired specimens, VCA-IgG was identified as positive in only 12.7% of the CSF and 100% of the serum specimens. This suggests that EBV VCA-IgG antibody, at least in most cases, cannot be transmitted through the blood–cerebral barrier. In the 20 pairs of samples with dual positives of VCA-IgG, 8 samples were from patients with CNS infections, 6 with encephalopathy, 2 with FUO and 1 with meningomyeloradiculitis, another 3 samples were from other hospitals in Beijing and the clinical details were unknown. As infections as well as other reasons of encephalopathy may increase the permeability of the blood–cerebral barrier, it was uncertain whether the VCA-IgG in CSF specimens in these 20 patients were produced locally or transmitted through the barrier except one 67 years old female patient with meningomyeloradiculitis (confirmed with diffuse large B cell lymphoma later), who had higher absorbance of VCA-IgG in the CSF than in the serum sample.

## Supporting information

S1 File Excel database of anti-EBV antibodies during 2013–2017 (anti-EBV.xlsx). (XLSX)
